# Antibodies to SARS-CoV-2 in dogs and cats, USA

**DOI:** 10.1080/22221751.2021.1967101

**Published:** 2021-08-18

**Authors:** Subarna Barua, Monirul Hoque, Folasade Adekanmbi, Patrick Kelly, Melinda Jenkins-Moore, Mia Kim Torchetti, Kelly Chenoweth, Theresa Wood, Chengming Wang

**Affiliations:** a College of Veterinary Medicine, Auburn University, Auburn, AL, USA; b Ross University School of Veterinary Medicine, Basseterre, St. Kitts & Nevis; c National Veterinary Services Laboratories, United States Department of Agriculture, Ames, IA, USA

**Keywords:** SARS-CoV-2, seroprevalence, ELISA, virus neutralization test, dogs, cats, USA

## Abstract

To provide more complete data on SARS-CoV-2 infections in dogs and cats in the U.S., we conducted a serosurvey on convenience serum samples from dogs (*n*=1336) and cats (*n*=956) collected from 48 states of the USA in 2020. An ELISA targeting the antibody against nucleocapsid identified eleven positive and two doubtful samples in cats, and five positive and five doubtful samples in dogs. A surrogate neutralization assay detecting antibodies blocking the attachment of the spike protein to ACE2 was positive with three of the ELISA positive and doubtful samples, and one of 463 randomly selected ELISA negative samples. These four positive samples were confirmed by SARS-CoV-2 virus neutralization testing. All were from cats, in New York, Florida, and New Jersey (*n*=2). The serosurvey results, one of the largest yet completed on dogs and cats globally, support the OIE and CDC positions that currently there is no evidence that pets play a role in the spread of SARS CoV-2 in humans.

## Introduction

The current Coronavirus Disease 2019 (COVID-19) pandemic is caused by the severe acute respiratory syndrome coronavirus 2 (SARS-CoV-2) which most likely originated from wildlife, in particular horseshoe bats (*Rhinolophus affinis*) or Malayan pangolins (*Manis javanica*), in China in 2019 [1,2]. Dogs and cats have their own coronaviruses [3], and the question arose early in the pandemic as to whether they can also be infected with the SARS-CoV-2. As dogs and cats often live in close association with people, if they were susceptible to infection, they might become clinically ill and act as sources of infection for people.

Recent studies have shown dogs have low susceptibility to SARS-CoV-2 infections. Experimentally infected animals can seroconvert but do not show clinical signs. Viral RNA might not be detected in oropharyngeal swabs, but rectal swabs can be positive for up to 6 days post-infection [4,5]. Although dogs in contact with experimentally infected dogs do not seroconvert [5], a small percentage of dogs in contact with people with COVID-19 (13%; 2/15) might become asymptomatically infected, and some can have low levels of viral RNA in nasal swabs for up to 9 days [6].

Cats appear to be more susceptible than dogs to SARS-CoV-2 infections and become clinically ill and even die following experimental infection. The infectious virus can be recovered from the upper and lower respiratory tracts for up to 10 days [5,7], and viral RNA can be detected in nasal washes for up to 9 days [5,7]. In-contact cats can become infected, most likely by respiratory droplet transmission [5,7,8]. Cats appear to develop robust immunity as they produce virus-neutralizing antibodies and are resistant to re-infection upon subsequent challenge [4]. Cats (12%; 6/50) in COVID-19 positive and close contact households can develop asymptomatic infections [9].

The World Organisation for Animal Health (OIE) and Centers for Disease Control (CDC) have produced statements indicating that currently there is no evidence that pets play a role in the spread of human infections with SARS-CoV-2 [10]. However, accumulating reports that cats and dogs are susceptible to SARS-CoV-2 has led to growing concerns of pets being abandoned by owners fearing they might be a source of infection. Although transmission from pets to humans has not yet been demonstrated, thousands of pets have been killed and abandoned [11]. Recommendations have been made that surveillance for SARS-CoV-2 in cats should be considered an adjunct to the elimination of COVID-19 in people [5]. In some areas, dogs and cats in COVID-19 positive and close contact households have been quarantined at home or in holding facilities until proven to be PCR negative [9]. Models have been developed that indicate abandoning cats into the environment could increase the risk of infection for people [12].

Recent limited studies from Europe and China suggest that natural infections of dogs and cats are infrequent, most commonly following exposure to COVID-19 patients [13,14]. Currently, there are 113 cases of SARS CoV-2 in companion animals reported in the U.S. (46 dogs and 67 cats), predominantly from COVID-19 positive households [15–17]. Confirmed cases in the U.S. are reported by the USDA to the OIE, and the CDC provides guidelines for protecting pets from infection and what to do if pets become infected [18–20].

Assays based on the detection of anti-SARS-CoV-2 IgG antibodies, which are typically detectable 7–21 days post-infection, can identify previous exposure even in asymptomatic individuals. ELISAs detecting the whole virus, nucleocapsid protein, and the receptor-binding domain (RBD) of the spike receptor protein have been widely used for the detection of antibodies against SARS-CoV-2 in humans [21,22]. Their usefulness in testing for infections in other species requires evaluation as there are a wide diversity of other coronaviruses in animals that might influence results. Although ELISAs can be used to rapidly process large numbers of samples in low-level security facilities, the laborious and slow SARS-CoV-2 virus neutralization test (VNT) which requires specialized biocontainment facilities (BSL3) is considered the “gold standard.” It is used by the CDC to classify a case as probably based on presumptive laboratory evidence [23].

Recently a surrogate neutralization assay (sVNT) was developed which detects neutralizing antibodies that block the attachment of the RBD of the spike protein to the ACE2 cell surface receptor [24]. This test uses an ELISA format which is simple and rapid to perform under BSL-2 conditions. It has high sensitivity and specificity compared with the 90% plaque reduction neutralization test (PRNT_90_) [25]. The VNT and sVNT are thus functional assays that detect a protective immune response, while the ELISAs can detect a greater range of antibodies and may, thus, give higher seroprevalences. For example, Hughes et al. [26] reported that only around half of the individuals who tested positive by ELISA for antibodies to the spike protein also had high VNT titers. Similarly, Zhang et al. reported that only 11 of 15 cats IgG positive in indirect ELISA targeting the RBD of the spike protein had SARS-CoV- 2 neutralizing antibodies detected by VNT [27].

To provide data on exposure of dogs and cats from around the USA to SARS-CoV-2, we tested convenience samples of sera from dogs and cats across 48 states. We initially screened the sera with a commercial double antigen sandwich ELISA kit before further testing selected samples with a commercial sVNT and a classical VNT method.

## Materials and methods

### Sera

Samples used in the study consisted of convenience samples of sera from apparently healthy dogs (*n*=1215) and cats (*n*=831) submitted to Auburn University College of Veterinary Medicine between March and November 2020 for rabies titer testing which is a requirement for international travel. We also tested sera from dogs (*n*=121) and cats (*n*=125) with clinical signs suggestive of hepatozoonosis and feline infectious peritonitis, respectively, that had been submitted for molecular diagnosis. No data was available on the COVID-19 status of the households from which the dogs and cats originated.

### SARS-CoV-2 double antigen ELISA

The ID Screen® SARS-CoV-2 Double Antigen ELISA (IDVet, rue Louis Pasteur, Grabels, France) was used to detect nucleocapsid-binding antibodies of SARS-CoV-2 in the dog and cat sera. The ELISA was performed according to the manufacturer’s instructions with an S/P % (sample to positive ratio) over 60% regarded as positive. Ratios from 50% to 60% were considered as doubtful, and those below 50% as negative.

### SARS-CoV-2 surrogate virus neutralization test

SARS-CoV-2 Surrogate Virus Neutralization Test (sVNT) Kits were purchased from GenScript (N.J., USA) and used according to the manufacturer’s instructions. The optical densities of the reactions of the test sera and the positive and negative controls supplied were read at 450 nm (OD_450_), and percentage inhibitions were calculated as follows: percent inhibition = (1 – sample O.D. value/negative-control O.D. value) ×100. Sera with percent inhibition values of ≥20% were regarded as positive while those giving lower values were considered to be negative [25].

### SARS-CoV-2 virus neutralization test

The virus neutralization tests were performed at the United States Department of Agriculture (USDA) National Veterinary Services Laboratories (NVSL), as described [28]. Briefly, 25 µL of two-fold serially diluted sera (for final dilutions of 1:8–1:512) were pre-incubated with 25 µL of 100 TCID50/ml of SARS-CoV-2 (2019-nCoV/USA-WA1/2020) in MEM-E containing 200UI/mL penicillin, 200 µg/mL streptomycin, 75 µg/ml gentamicin sulfate and 6 µg/mL Amphotericin B for 60 min at 37°C with 5% CO2. Each serum sample was tested in duplicate in 96-well plates. At one hour post-infection, 150 µl of Vero 76 cells were added to the virus-serum mixtures. The neutralization titers were determined at three days post-infection. The titer was recorded as the reciprocal of the highest serum dilution that provided 100% neutralization of the reference virus, as determined by visualization of cytopathic effect. Neutralizing titers of 8 and 16 were considered suspect in the absence of other positive tests; titers greater than 16 were considered seropositive. Samples positive for SARS-CoV-2 at NVSL were reported to the OIE.

## Results

With the I.D. Screen ELISA, six sera from cats were found to be positive, two were found to be doubtful, and 948 were negative (Table 1). With the I.D. Screen ELISA, five sera from dogs were found to be positive, five were found to be doubtful, and 1326 were negative (Table 2). None of the sera from the 125 cats with suspected FIP were positive in the I.D. Screen ELISA (tend to delete this sentence). Two of the 121 dogs (ID Numbers: 1098, 2013) with suspected *Hepatozoon americanum* were positive in the IDVet Screen ELISA but subsequently were negative in both the sVNT and VNT.

**Table 1.** Eleven sera tested for cats by I.D. Screen ELISA, sVNT and VNT in this study.

Sample ID	Species	Breed	Sex	Age	IDVet Screen S/*P* ratio and O.D. value	GenScript sVNT inhibition value; OD value	VNT titer
2538	Cat	Munchkin	Male	20 M	292.1%; 2.39	97.3%; 0.044	1:256
2903	Cat	DSH	Female	7 Y	240.7%; 2.34	97.4%; 0.043	1:128
2620	Cat	DSH	Female	2 Y	220.4%; 2.24	96.9%; 0.046	1:32
2410	Cat	DSH	Female	2Y2M	89.3%; 0.92	−2.1%; 1.67	negative
3213	Cat	DSH	Female	5 Y	70.4%; 0.77	−9.1%; 1.78	negative
3577	Cat	DSH	Female	2 Y	63.5%; 0.71	−10.8%; 1.81	negative
3329	Cat	Ragdoll	Female	10 M	58.8%; 0.68	−7.4%; 1.75	negative
1960	Cat	Ragdoll	Female	14 M	54.2%; 0.59	0.2%; 1.63	negative
2797	Cat	DSH	Male	6 Y	49.2%; 0.55	97.2%; 0.050	1:32
3372	Cat	Bengal	Female	5 Y	41.7%; 0.47	−2.9%; 1.68	negative
2931	Cat	DSH	Female	4 Y	41.2%; 0.44	0.3%; 1.63	negative

**Table 2.** Fifteen sera tested for dogs by I.D. Screen ELISA, sVNT and VNT in this study.

Sample ID	Species	Breed	Sex	Age	IDVet Screen S/*P* ratio and O.D. value	GenScript sVNT inhibition value; OD value	VNT titer
1098	Dog	Pyrenees Mix	Male	8Y	129.6%; 1.21	−0.4%; 1.71	negative
107	Dog	Labrador Retriever	Female	5Y	119.6%; 1.16	7.9%; 1.51	negative
1243	Dog	Shih Tzu	Male	7Y	109.4%; 0.99	−6.8%; 1.74	negative
2013	Dog	Miniature poodle	Female	4Y	101.5%; 1.06	−1.8%; 1.66	negative
2075	Dog	Terrier Mix	Male	1Y	69.3%; 0.62	−0.5%; 1.64	negative
2166	Dog	Shiba Inu	Male	7 Y	59.5%; 0.63	−4.0%; 1.70	negative
2617	Dog	Border Collie	Female	4 Y	59.5%; 0.64	−9.5%; 1.75	negative
3180	Dog	Collie Rough Coat	Male	6 Y	54.5%; 0.61	−1.2%; 1.65	negative
1187	Dog	Karelian Bear Dog	Female	12 Y	53.3%; 0.37	1.7%; 1.60	negative
351	Dog	Mixed breed	Male	3 Y	52.0%; 0.48	−3.9%; 1.70	toxic
3496	Dog	Miniature Schnauzer	Male	10 M	47.5%; 0.49	0.3%; 1.63	negative
463	Dog	Mixed breed	Female	10 M	44.2%; 0.42	−0.1%; 1.63	toxic
462	Dog	American domestic shorthair	Female	5 M	43.0%; 0.41	−5.8: 1.73	toxic
2685	Dog	Basenji Mix	Female	21 M	38.3%; 0.43	−12.7%; 1.84	negative
3408	Dog	Border Collie	Male	1Y 4M	37.6%; 0.43	−3.8%; 1.70	negative

Twenty-six sera with the highest S/P ratios in the I.D. Screen ELISA (11 positive, seven doubtful, and six negative samples) and 434 randomly selected negative samples were further tested with the sVNT. Three of the eleven I.D. Screen ELISA positive samples and one of the 434 negative samples gave positive results in the sVNT (Table 1). All the I.D. Screen ELISA doubtful samples were negative in the sVNT.

When the above 26 sera with the highest S/P ratios in the I.D. Screen ELISA were further tested by VNT at the NVSL, only the samples positive in the sVNT were positive (Table 1). The sample the sVNT identified as positive amongst the 434 found negative by the I.D. Screen ELISA also tested positive in the VNT (Table 1). Three samples showed toxic effects by VNT. One had given a doubtful result in the I.D. Screen ELISA (S/P% = 49.2%) but was negative in the sVNT. The remaining two were negative in both the I.D. Screen ELISA and the sVNT.

Although the VNT positive results represent only a single point in time, they are suggestive of infection with SARS-CoV-2 and meet the USDA case definition for confirmed SARS-CoV-2 cases [23]. While the VNT titers of the four confirmed cases ranged from 1:32 to 1:256, all four had high inhibition values with the sVNT (around 97%) (Table 1). Three of the four VNT positive cases also had high S/P ratios (>220%) in the I.D. Screen ELISA. The remaining sample (ID Number 2620), with a VNT titer of 1:32, had an S/P ratio of 48.2% in the I.D. Screen ELISA, just below the value of 50%–60% indicative of a doubtful result.

All the VNT positive sera were from cats: one from N.Y. (ID number 2538), one from Florida (ID 2797) and two from New Jersey (ID 2620 and ID 2903).

## Discussions

Overall, regarding the results of negative samples, there was a relatively good correlation between the performances of the IDVet Screen ELISA and the sVNT with the tests agreeing on 433 of the 434 samples. However, of the 11 samples found positive for antibodies to the nucleocapsid proteins in the IDVet Screen ELISA, only 3 had neutralizing antibodies against the RBD of the spike protein as demonstrated by a positive VNT. There are a variety of coronaviruses that can infect dogs and cats and, in general, nucleocapsid proteins of coronaviruses are relatively conserved with significant antigenic cross-reactivity [29,30]. There is thus the possibility that the IDVet Screen ELISA positives were most likely due to exposure to other animal coronaviruses.

The cat that was negative by the IDVet Screen ELISA but positive in both the GenScript sVNT and VNT most likely had been exposed to the SARS CoV-2 but not developed neutralizing antibodies against the nucleocapsid. The presence of antibodies against the spike protein in the absence of antibodies against nucleocapsid proteins is not uncommon in people (Tehrani et al., 2020; Liu et al., 2021).

Despite the USA being one of the countries most seriously affected by the COVID-19 pandemic, we found none of the convenience serum samples of dogs (0/1336) we studied were positive for antibodies to SARS CoV-2. Further, only very few of our convenience samples from cats (0.4%; 4/956) had serological evidence of infection. The SARS-CoV-2 positivity rate of pets in households with unknown COVID-19 status in the U.S. would thus appear to be very low (0.17%; 4/ 2289 animals).

Our serosurvey of cats and dogs for antibodies to SARS CoV-2 adds to the growing body of information from smaller studies on infections in pets worldwide [14,27,28,31–36]. Studies have shown that cats experimentally infected with ∼10^5^ PFU can readily transmit SARS-CoV-19 to naïve cats when placed in continuous and close confinement [4,5,8]. In contrast, naïve dogs closely confined with experimentally infected dogs do not become infected [5]. Although animal-to-human transmission of SARS COV-2 occurs in minks [37], there is currently no evidence that dogs and cats can transmit infections to people [13]. Instead, reports on dogs and cats in households with people suffering from COVID-19 [13] indicate that pets are infected by people. Such transmission appears to be low, however, with PCR studies showing only low positivity rates in pets in COVID-19 positive households. For example, in Spain, only 1/8 (12%) cats and 0/12 dogs tested positive in COVID-19 positive households [32], while in Hong Kong, only 6/50 (12%) cats [9] and 2/15 (13%) dogs were positive [6]. Also, transmission is not inevitable, and in a report of 9 cats and 12 dogs living among 20 French veterinary students (2 with confirmed COVID-19 and 11 with clinical signs), none of the pets were seroconverted [36].

The seroprevalence we found in cats in the U.S. is very similar to that of 0.69% (6/920) and 0.76% (1/131) reported in the only other large-scale serosurveys carried out to date, in Germany [14] and Croatia [35], respectively. Both surveys were carried out on cats from households with unknown COVID-19 status in the early stages of the pandemic when the incidence of human infection was likely still relatively low. Our study was conducted until November 2020 and thus included the first COVID-19 wave of infection and the initial phases of the second wave when pet exposure would appear more likely. Other smaller surveys of cats with an unknown history of exposure have revealed seropositive animals in China (0%, 0/86, 34; 15%,15/102; 27), France (6%, 1/16; 30), and Italy (5.1%; 2/39; 31) for example.

A limitation of our study is that we had no information on the COVID status of the households containing the animals we studied. We anticipate, however, that as the sera we tested were submitted for rabies titers there was the anticipation of international travel which would not be expected in households with active COVID-19. We suspect, then, that most of the animals we studied were from households with no history of COVID-19 patients and that have had households with active human infections might provide higher prevalences. Although seroprevalences are difficult to compare directly because of differences in serological techniques used in experiments, dogs and cats in contact with COVID-19 patients have been reported to be eight times more likely (relative risk 8.1) to be seropositive than those in homes of unknown exposure [30]. Cat seroprevalences in households with COVID-19 patients have been reported from Italy (4.5%, 1/22; 31), France (0%; 0/9; 36; 23.5%, 8/34; 30), and the US (4/ up to 34; 28), for example.

In conclusion, our data indicate dogs and cats in the U.S. appear to be infrequently infected with SARS CoV-2, and this supports other evidence that companion animals are not a significant source of human infection. A recent study [35] has shown animal health workers and veterinary laboratory personnel in contact with animals and their products are no more likely to become seropositive than workers with no animal contact. Companion animals provide multifaceted health benefits to their owners, including increased emotional well-being, significant stress reduction, and increased physical activity. Such benefits are particularly important to senior citizens during isolation and stress induced by the COVID-19 pandemic. Media should thus be encouraged to refrain from emotive reporting on the role of pets in COVID-19. The current OIE [38], CDC [10], and AVMA [39,40] recommendations relating to companion animal infections should be followed closely. Companion animal veterinarians need to be alerted to the possibility, albeit low, of SARS-CoV-2 in their patients and the most appropriate treatments and methods to prevent spread of infection to other animals and people in the household [40].

## Supplementary Material

Supplementary_Table_1.docx
