# Supplementary material for: Antibodies to SARS-CoV-2 in dogs and cats, USA
Source: Emerg Microbes Infect. 2021 Aug 18;10(1):1669–74. doi: 10.1080/22221751.2021.1967101 (PMC8381919; doi:10.1080/22221751.2021.1967101)
Supplement: Supplementary_Table_1.docx [file TEMI_A_1967101_SM2624.docx]

**Supplementary Table 1. Sera samples used in this investigation**

| **State** | **Number of samples submitted** | | | **Tested positive** | | | | | |
| --- | --- | --- | --- | --- | --- | --- | --- | --- | --- |
|  |  |  |  | **IDVet (N=2117)** | | **VNT (N=26)** | | **sVNT (N=460)** | |
|  | **Total** | **Canine** | **Feline** | **Canine** | **Feline** | **Canine** | **Feline** | **Canine** | **Feline** |
| Alabama | 123 | 91 | 32 | 1 |  |  |  |  |  |
| Alaska | 0 | 0 | 0 |  |  |  |  |  |  |
| Arizona | 12 | 7 | 5 |  |  |  |  |  |  |
| Arkansas | 24 | 7 | 17 |  |  |  |  |  |  |
| California | 439 | 208 | 231 | 1 | 1 |  |  |  |  |
| Colorado | 20 | 17 | 3 |  |  |  |  |  |  |
| Connecticut | 13 | 2 | 11 |  |  |  |  |  |  |
| Delaware | 6 | 2 | 4 |  |  |  |  |  |  |
| District of Columbia | 0 | 0 | 0 |  |  |  |  |  |  |
| Florida | 177 | 136 | 41 |  | 1 |  | 1 |  | 1 |
| Georgia | 121 | 93 | 28 |  |  |  |  |  |  |
| Hawaii | 59 | 45 | 14 |  |  |  |  |  |  |
| Idaho | 14 | 7 | 7 |  |  |  |  |  |  |
| Illinois | 47 | 26 | 21 |  |  |  |  |  |  |
| Indiana | 18 | 6 | 12 |  | 1 |  |  |  |  |
| Iowa | 12 | 7 | 5 |  |  |  |  |  |  |
| Kansas | 7 | 3 | 4 |  |  |  |  |  |  |
| Kentucky | 7 | 6 | 1 |  |  |  |  |  |  |
| Louisiana | 15 | 12 | 3 |  |  |  |  |  |  |
| Maine | 2 | 2 | 0 |  |  |  |  |  |  |
| Maryland | 8 | 3 | 5 |  |  |  |  |  |  |
| Massachusetts | 81 | 34 | 47 |  |  |  |  |  |  |
| Michigan | 34 | 26 | 8 |  |  |  |  |  |  |
| Minnesota | 13 | 5 | 8 |  |  |  |  |  |  |
| Mississippi | 35 | 30 | 5 | 1 |  |  |  |  |  |
| Missouri | 13 | 10 | 3 |  |  |  |  |  |  |
| Montana | 3 | 3 | 0 |  |  |  |  |  |  |
| Nebraska | 4 | 3 | 1 |  |  |  |  |  |  |
| Nevada | 9 | 5 | 4 |  |  |  |  |  |  |
| New Hampshire | 9 | 1 | 8 |  |  |  |  |  |  |
| New Jersey | 12 | 8 | 4 | 1 |  |  | 2 |  | 2 |
| New Mexico | 5 | 5 | 0 |  |  |  |  |  |  |
| New York | 304 | 141 | 163 |  | 2 |  | 1 |  | 1 |
| North Carolina | 25 | 9 | 16 |  |  |  |  |  |  |
| North Dakota | 0 | 0 | 0 |  |  |  |  |  |  |
| Ohio | 52 | 29 | 23 |  |  |  |  |  |  |
| Oklahoma | 16 | 14 | 2 |  |  |  |  |  |  |
| Oregon | 5 | 2 | 3 |  | 1 |  |  |  |  |
| Pennsylvania | 90 | 66 | 24 |  |  |  |  |  |  |
| Rhode Island | 5 | 3 | 2 |  |  |  |  |  |  |
| South Dakota | 0 | 0 | 0 |  |  |  |  |  |  |
| South Carolina | 21 | 9 | 12 |  |  |  |  |  |  |
| Tennessee | 38 | 35 | 3 | 1 |  |  |  |  |  |
| Texas | 91 | 59 | 32 |  |  |  |  |  |  |
| Utah | 7 | 7 | 0 |  |  |  |  |  |  |
| Vermont | 4 | 2 | 2 |  |  |  |  |  |  |
| Virginia | 37 | 20 | 17 |  |  |  |  |  |  |
| Washington | 56 | 24 | 32 |  |  |  |  |  |  |
| West Virginia | 2 | 1 | 1 |  |  |  |  |  |  |
| Wisconsin | 11 | 5 | 6 |  |  |  |  |  |  |
| Wyoming | 3 | 1 | 2 |  |  |  |  |  |  |
| Unknown | 8 | 5 | 3 |  |  |  |  |  |  |
| **Total** | **2117** | **1242** | **875** | **5** | **6** | **0** | **4** | **0** | **4** |
